# Clinical Characteristics of Adults Living with a Spinal Cord Injury Across the Continuum of Care: A Population-Based Cross-Sectional Study

**DOI:** 10.3390/jcm14093060

**Published:** 2025-04-29

**Authors:** Matteo Ponzano, Anja Declercq, Melissa Ziraldo, John P. Hirdes

**Affiliations:** 1 School of Health and Exercise Science, University of British Columbia, 1238 Discovery Ave, Kelowna, BC V1V 1V7, Canada; 2 International Collaboration on Repair Discoveries (ICORD), Blusson Spinal Cord Centre (BSCC), University of British Columbia, Vancouver, BC V5Z 1N1, Canada; 3 LUCAS–Centre for Research, KU Leuven, 3000 Leuven, Belgium; anja.declercq@kuleuven.be; 4 School of Public Health Sciences, University of Waterloo, Waterloo, ON N2L 3G1, Canada; mziraldo@uwaterloo.ca (M.Z.); hirdes@uwaterloo.ca (J.P.H.); 5 Center for Sociological Research, Faculty of Social Sciences, KU Leuven, 3000 Leuven, Belgium

**Keywords:** Paraplegia, Tetraplegia, healthcare services, long-term care, clinical outcomes

## Abstract

**Background/Objectives**: People living with a spinal cord injury (PwSCI) present numerous complications at a systemic level that negatively impact their physical and mental health as well as their quality of life. The purpose of this study was to describe the clinical profile of PwSCI living in nursing homes (NHs), Complex Continuing Care Systems (CCCs), home care (HC), and inpatient mental health facilities (MHs) in nine Canadian provinces and territories. **Methods**: We analyzed data collected with the following assessment tools: Resident Assessment Instrument (RAI) Minimum Data Set (RAI-MDS 2.0), RAI–MH, RAI-HC, Cognitive Performance Scale, Activities of Daily Living (ADL) Hierarchy Scale and impairments in instrumental ADLs (IADLs), Pain Scale, Changes in Health, End-Stage Disease, Signs, and Symptoms (CHESS) Scale, Depression Rating Scale, and Deafblind Severity Index (DBSI). We reported counts (*n*) and percentages (%) and performed Chi-square tests with a Bonferroni correction to determine the statistical significance of the differences in frequencies within and between care settings. **Results**: We identified 13,136 PwSCI, predominantly males and younger than comparison groups. PwSCI presented fewer comorbidities but reported higher pain than comparison groups. Almost all of the PwSCI in NHs (99.4%) and CCCs (98.9%) needed assistance to perform ADLs. **Conclusions**: The prevalence of comorbidities and impairments following SCI varies based on the clinical setting. The present clinical profile of PwSCI will inform interventions to improve health of PwSCI across the continuum of care.

## 1. Introduction

Approximately 86,000 people live with spinal cord injury (PwSCI) in Canada, with an estimated incidence of 3675 new cases every year [1]. A retrospective study in 831 individuals with a traumatic SCI showed that the mean age of injury increased from 39.1 ± 15.9 years in 2002 to 52.1 ± 19.0 years old in 2010 [2]. Moreover, current evidence indicates that the highest incidence of traumatic SCI has been observed in persons 55 years or older [2]. The increasing age of the SCI population and the numerous complications at a systemic level prompted an exploration of the clinical characteristics of this population across the continuum of care, with the ultimate goal to design more effective management plans to improve quality of life and prevent further comorbidities.

The level and the completeness of the lesion determine the entity of impairments and secondary complications after SCI. Chronic pain of unspecified etiology and neuropathic pain are present in approximately 60% of the SCI population [3,4], and have a negative impact on mental health [5,6]. Suboptimal mental health further decreases social participation, physical health, and functioning among PwSCI. Poor mental health also increases the risk of secondary health complications [7], substance abuse [8], and clinical psychological disorders (e.g., anxiety and depressive symptoms, post-traumatic stress) [9]. Mortality rates are higher in the first year post-injury, and infections, along with cardiovascular and pulmonary complications, are the principal causes of death in PwSCI [10,11]. The entity of cardiovascular complications is related to the level of the injury [12], and it has been estimated that 68% of persons with American Spinal Injury Association (AIS) A and B SCI will develop arterial hypotension, and up to 16% will suffer a cardiac arrest [13].

interRAI aims to improve health systems’ capacity to assess and report on clinical outcomes, to facilitate data sharing and interpretations across health settings. interRAI instruments were designed and tested to collect person-level data in a wide array of domains, including but not limited to physical functioning, cognition, mood and behavior, social participation, health conditions, and medications [14,15,16]. These assessment tools have been implemented in over 35 countries, and are mandated by governments in several countries, including many US states and Canada. In Canada, interRAI assessment instruments are routinely used in nursing homes (NHs), Complex Continuing Care hospitals/units (CCCs), home care (HC), and inpatient mental health facilities (MHs). The Innovations in Data, Evidence, and Applications for Persons with Neurological Conditions project was created to describe sociodemographic and clinical characteristics, impairments, and limitations of adults living with neurological conditions including multiple sclerosis [17], motor neuron disease [18], Alzheimer and related dementias [19], and epilepsy [20] in NHs, CCCs, HC, and MHs. The aim of the present study was to establish a clinical profile of Canadian PwSCI across the continuum of care in nine Canadian provinces and territories.

## 2. Methods

Data were obtained through an existing license agreement between interRAI and the Canadian Institute for Health Information (CIHI). Ethics approval for this project was received from the Office of Research Ethics at the University of Waterloo (ORE#30372). The present manuscript is reported according to the Strengthening the Reporting of Observational Studies in Epidemiology (STROBE) Statement [21].

### 2.1. Data Sources

The present paper reports on data obtained in NHs, CCCs, HC, and MHs in nine Canadian provinces and territories that have implemented interRAI assessments including the Resident Assessment Instrument (RAI) Minimum Data Set (RAI-MDS 2.0), the Resident Assessment Instrument—Mental Health (RAI–MH), or the Resident Assessment Instrument Home Care (RAI-HC). All possible sources of information are used to complete the assessments, including interviews with and observation of the person, discussions with other staff and family, and hospital chart review.

### 2.2. Nursing Homes

The NH cohort includes quarterly and full RAI-MDS 2.0 assessments conducted in Ontario (n = 635 facilities) and Nova Scotia (n = 8 facilities) from 1 July 2003 to 31 March 2023, British Columbia (n = 126 facilities) from 1 July 2006 to 31 March 2023, and in Manitoba (n = 38 facilities part of the Winnipeg Regional Health Authority, accounting for 60% of the Manitoba population), Newfoundland (n = 7 facilities), Saskatchewan (n = 168 facilities), and Yukon Territory (n = 2 facilities) from 1 July 2008 to 1 March 2023.

### 2.3. Complex Continuing Care Systems

CCCs provide hospital-based long-term complex medical care, geriatric assessment and rehabilitation, psycho-geriatric care, palliative care, and respite care [22]. Usually, CCC patients are more medically complex, and stays are generally shorter than in NHs. The CCC cohort includes quarterly and full RAI-MDS 2.0 assessments conducted in 173 CCC units/hospitals in Ontario from 10 April 1996 to 31 March 2023 and in 173 CCC units/hospitals in Manitoba from 1 July 2008 to 31 March 2023.

### 2.4. Inpatient Mental Health Facilities

The MH cohort includes RAI–MH assessments conducted at admission, every 90 days thereafter, and upon discharge in 55 hospitals with inpatient psychiatric units/beds and 13 specialty psychiatric hospitals in Ontario from 1 October 2005 to 31 March 2023. Coverage is considered census-level, or 100% of Ontario adult inpatient mental health beds.

### 2.5. Home Care

The HC cohort includes assessments conducted twice yearly (every six months) using the RAI-HC. Coverage is considered census-level, as it includes demographic, clinical, and functional data, as well as service use information for persons expected to be on service for ≥60 days from all the 14 regional home care agencies in Ontario.

### 2.6. Patient Populations

#### 2.6.1. Persons with SCI

Persons with an SCI diagnosis were identified based on pick list item responses on the RAI-MDS 2.0, RAI-HC, and free-text International Statistical Classification of Diseases and Related Health Problems, Tenth Revision, Canada (ICD-10-CA) fields on the RAI-MH instrument. Clinicians are instructed to complete these assessments using all sources of information available. Information can be obtained through observation, patient interviews, medical records (i.e., physician orders, laboratory data, medication records, and care plans), and consultation with family members or the attending physician. Instruments relying on pick list responses achieved very high sensitivity (90–94%), specificity (99–100%), and interrater agreement (kappa = 0.76–0.84) [23]. The RAI-MH, which relies on ICD-10-CA code responses, also performed well with a sensitivity of 77%, specificity of 100%, and kappa = 0.61 [23].

#### 2.6.2. Comparison Group

The comparison group included individuals without a diagnosis of SCI, other neurological conditions, or stroke who were administered the same assessments as the SCI groups in the four care settings. Conditions such as neuropathy, migraine, and tension headache are not reported in interRAI assessments; therefore, individuals with these conditions may be present in any of the study groups.

### 2.7. Outcome Measures

Patients’ medical conditions were recorded using the RAI-MDS 2.0, RAI-HC, and RAI-MH instruments, which assess common outcomes using validated clinical scales across several domains (such as physical functioning, cognition, mood and behavior, social functioning, other comorbidities and diseases, health services and medication utilization). The Cognitive Performance Scale (CPS) is a brief measure of cognitive impairment that ranges from 0 (intact) to 6 (very severely impaired) [24,25,26]. The Activities of Daily Living Hierarchy Scale is a measure of functional performance that ranges from 0 (no impairment) to 6 (total dependence) based upon the ability to complete early and late loss activities of daily living (ADLs) [27], and we collected data on whether instrumental ADLs (IADLs) such as meal preparation, managing finances, managing medications, and transportation were impaired or not. The pain scale is a visual analog scale that ranges from 0 (no pain) to 3 (excruciating pain) and is used to assess general pain levels [28]. The Changes in Health, End-Stage Disease, Signs, and Symptoms (CHESS) Scale is a measure of health instability that ranges from 0 (no health instability) to 5 (very high health instability) and is predictive of mortality [29,30]. The Depression Rating Scale (DRS) is an instrument to screen for depression, that ranges from 0 to 14, with scores ≥ 3 indicating depressive disorders [31]. The Deafblind Severity Index (DBSI) is a measure of sensory disability that ranges from 0 (both hearing and vision intact) to 5 (both hearing and vision severely impaired) [32].

### 2.8. Data Analysis

We extracted demographic characteristics from the items and scales in the appropriate instrument to the each of the four care settings (RAI-MDS 2.0, RAI-HC, and RAI-MH), and presented them as categorical outcomes reported as counts (n) and percentages (%). We included in the analysis clinical and demographic data obtained in the most recent assessment of each individual. We performed Chi-square tests to determine the statistical significance of the differences in proportion within each care setting and between HC, NHs, and CCCs. To account for the high number of statistical tests performed, we applied a Bonferroni correction that yielded an adjusted α of 0.05/105 = 0.0005.

To estimate the point prevalence of SCI in each of the four care settings, a prevalence sample comprising Ontario patients receiving care in one of the four care settings on 31 March 2023 (index date) was created. This index date was selected because there was complete interRAI assessment coverage across the four care settings in Ontario at that time. For patients in NH, CCCs, and MH settings, facility admission and all-cause discharge dates were used to determine inclusion in the prevalence sample. HC patients in Ontario may remain on service for long periods without actively receiving home care services (e.g., home health aide or nursing visits). To restrict the prevalence sample to patients actively receiving home care services, only patients with a home care referral date before the index date and a RAI-HC assessment completed 180 days before or after the index date were included. Where all-cause discharge dates were available for home care patients, this information was also used to determine inclusion in the secondary sample. All analyses were conducted using SAS, version 9.2 (SAS Institute Inc., Cary, NC, USA).

## 3. Results

We identified 13,136 PwSCI across the four care settings. PwSCI were mostly males in all the care settings and more common in CCCs (n = 7646; females: 45.3%; age ≥ 65 years: 54.9%; paraplegia: 50.7%), followed by NHs (n = 5146; females: 47.1%; age ≥ 65 years: 67.5%; paraplegia: 55.7%), HC (n = 312; females: 32.1%; age ≥ 65 years: 41.45%), and MHs (n = 32; females: 34.4%; age ≥ 65 years: 12.5%). All of the within- and between-setting comparisons were statistically significant (*p* < 0.0001) unless otherwise stated (Table 1).

### 3.1. Comorbidities, Medical Treatments, and Other Clinical Measures

PwSCI presented fewer comorbidities than comparison groups (Table 2). The majority of PwSCI reported minimal-to-low health instability (HC: 58.0%, NHs: 47.7%, and CCCs: 44.3%), and no-to-slight visual or hearing impairment (HC: 91.0%, NHs: 78.4%, CCCs: 81.4%). In the CCCs cohort, 76.0% and 45.1% reported bowel and bladder incontinence, respectively; similarly, bowel and bladder incontinence were reported by 78.5% and 63.4% of PwSCI in NHs. In the HC cohort, 22.4% and 26.0% reported bowel and bladder incontinence, respectively. In the MH cohort, most PwSCI (68.8%) showed no evidence of health instability, and 15.6% and 18.8% had bowel and bladder incontinence, respectively. In NHs, 53.9% of PwSCI were diagnosed with any psychiatric disorders, compared to 44.0% in CCCs and 40.1% in HC. In the CCC cohort, 49.8% of PwSCI reported trouble swallowing, compared to and 42.4% in NHs, and 8.7% in HC; none of the PwSCI in MHs reported trouble swallowing (*p* = 0.6597). The number of PwSCI that reported pressure ulcers was higher in HC (26.9%) than CCCs (21.7%) and NHs (14.2%), and more PwSCI needed wound care in CCCs (62.8%) than NHs (48.3%) and HC (28.5). All of the within- and between-setting comparisons were statistically significant (*p* < 0.0001) unless otherwise stated (Table 2).

### 3.2. Pain, Mobility, Falls, Fractures, Activity of Daily Living

PwSCI showed higher pain than the comparison groups, and pain levels varied across settings. The majority of PwSCI reported mild-to-moderate pain levels in CCCs (56.7%), HC (49.7%), NHs (41.2%), and MHs (37.5%, Table 3). Severe-to-excruciating pain was reported by 33.7% of PwSCI in HC, 12.1% in CCCs, 5.3% in NHs, and 6.3% in MHs. No pain was reported by 53.5% of PwSCI in NHs, 31.3% in CCCs, 26.3% in MHs, and 16.7% in HC. PwSCI were more likely to use a wheelchair as a primary mode of mobility in NHs (86.0%), CCCs (70.0%), and HC (60.6%). The highest number of PwSCI who experienced at least one fall in the previous 90 days was recorded in HC (27.9%), followed by MHs (9.4%). The proportion of PwMS that experienced a fall in CCCs and NHs were 5.6% and 4.1%, respectively. In NHs, 17.2% of PwSCI were diagnosed with osteoporosis, 14.4% in HC (*p* = 0.0042), and 8.9% in CCCs. In the MH and HC cohorts, 71.9% and 37.2% were independent in performing ADLs, while almost all of the PwSCI in NHs (96.1%) and CCCs (95.5%) were totally dependent with regard to performing ADLs. All of the within- and between-setting comparisons were statistically significant (*p* < 0.0001) unless otherwise stated (Table 3).

### 3.3. Mental Health, Cognition, and Psychotropic Medications

Most PwSCI reported the severe-to-very severe cognitive impairment in CCCs (33.8%) and NHs (28.8%), while 56.1% of PwSCI in HC and 62.5% in MHs reported no cognitive impairment. The differences in DRS scores were not statistically significant across sectors (*p* = 0.25), with the majority of PwSCI that showed depressive symptoms, while we detected statistically significant differences across sectors in anxiety symptoms (*p* = 0.0002) and in the use of both anti-depressants and anti-anxiety medications. All of the other within- and between-setting differences in proportion were statistically significant (*p* < 0.0001; Table 4).

## 4. Discussion

The present study established the clinical profile of PwSCI across the continuum of care. We analyzed data from over 10% of the PwSCI living in Canada and provided a comprehensive description of the different health conditions in PwSCI across the care settings. Our comparisons between PwSCI and persons without any neurological disorders allowed us to report on the differences between persons with and without SCI in each care setting. Further, the clinical profile of PwSCI varies extremely across the continuum of care, so we cannot assume that each issue occurs with the same magnitude or severity for SCI groups in different care settings. Therefore, an SCI diagnosis alone is relatively uninformative, and the complex array of symptoms and needs highlight the importance of comprehensive assessments such as the interRAI systems, with the ultimate goal of providing relevant information for clinicians in terms of prevention and management of health issues in PwSCI.

Pain is one of the most prevalent and debilitating consequences of SCI, as it has been estimated that at least 60% of PwSCI live with chronic pain [4]. The level of pain varied across settings: the majority of PwSCI in CCCs, HC, and MHs reported mild-to-moderate or excruciating pain, while over 50% of PwSCI in NHs reported no pain. Our findings are in line with previous systematic reviews in PwSCI [33] and in NH residents [34], which observed a prevalence of pain ranging between 20% and 90%. The high variability is not surprising, as there are many concurrent factors that can originate and/or exacerbate pain. Biopsychosocial approaches conceptualize pain as a combination of physiological, psychological (emotional, cognitive, and behavioral), and social (interaction with others) factors that may influence both the perception and the management of pain [35,36]. The CanPain SCI clinical practice guidelines for rehabilitation and management of neuropathic pain after SCI recommend an interprofessional, patient-centered, and biopsychosocial management of neuropathic pain [37]. Our data showed that half of the PwSCI in CCCs and NHs received physical or occupational therapy in the previous week, and 70% to 90% of PwSCI in such sectors received a physician visit in the previous 14 days, but only 1% to 11% received counselling by a social worker or a psychologist in the previous 7 days. Therefore, the care setting is a major determinant of whether PwSCI can access rehabilitation services. In HC, NHs, and CCCs, PwSCI have average longer stays and thus have different care trajectories than the comparison groups. Furthermore, in CCCs, PwSCI present with both ADL and cognitive impairments, which may explain the need for a more complex hospital-based long-term medical care. The adoption of biopsychosocial, standardized protocols for the management of chronic pain across the continuum of care, along with training and guidance for healthcare professionals, may improve pain perception and management in PwSCI.

Pressure ulcers are another common and debilitating secondary complication of SCI. The US National Pressure Ulcer Advisory Panel (NPUAP) defines pressure injuries as localized damage to the skin and underlying soft tissue, commonly over a bony prominence or related to a medical or other device [38]. The global prevalence of pressure ulcers in PwSCI is estimated to be around 30% [39], and up to 95% of PwSCI will experience a pressure ulcer during their lifetime [40,41,42]. Pressure injuries cause several physical and mental health complications, and account for 7–8% of premature deaths in people with spinal cord injury [43,44,45]. People with complete SCI have greater odds of developing a pressure ulcer compared to people with incomplete SCI [46], and the high number of people with incomplete SCI in our population may explain the lower prevalence of pressure ulcers compared to global estimates. While interRAI assessment tools only report on non-specific surgical wound care received in the previous seven days, the prevalence of pressure ulcers and the percentage of people receiving wound care is higher among PwSCI compared to the comparator group, and that may denote a proper management of pressure ulcers.

Bowel dysfunction is one of the most important secondary complications experienced by PwSCI [47], with an estimate of 8% of PwSCI experiencing daily faecal incontinence [48]. Bowel incontinence was more prevalent in PwSCI compared to the comparison group in all care settings, exceeding 75% of PwSCI in CCCs and NHs, in line with a previous Danish cohort study, which reported a prevalence of faecal incontinence up to 75% [49]. Conversely, the percentage of people experiencing bladder incontinence was lower in PwSCI than the comparison group in all the care settings. The prevalence of urinary incontinence was highly heterogeneous across setting, ranging from 19% in MHs to 63% in CCCs. Previous studies in PwSCI reported a 43–49% prevalence of bladder incontinence [50,51], and 27% and 13% of participants experienced urinary incontinence daily and weekly, respectively [51]. Bowel and bladder dysfunction can have a negative effect on mental health, and it has been showed that depressive symptoms are worse in PwSCI with more severe incontinence [52,53]. PwSCI face many physical, emotional, and social challenges and stressors that increase symptoms of depression and anxiety and reduce satisfaction with life [9,54]. Therefore, considering the large prevalence of mental illness among PwSCI, it is not surprising that we did not detect any statistically significant difference in depressive symptoms between HC, NHs, and CCCs. Further, the younger average age of PwSCI compared to people without an SCI may explain the fewer comorbidities observed among PwSCI than in the comparison groups. Clinicians should take into account the different experiences, values, and preferences of PwSCI when developing treatment and rehabilitation plans, as they may be different from those of comparison groups who predominantly include older adults in the four care settings.

Osteoporosis, especially below the neurological level, is a common consequence of SCI [55,56,57]. It has been estimated that PwSCI lose up to 80% and 30% of tibial trabecular and cortical bone, respectively [58], with a reported ongoing bone loss of 0.45% per month at the tibial epiphysis as late as ten years post SCI [59]. However, the prevalence of osteoporosis was low across all the care settings and, in NHs and CCCs, the number of PwSCI with osteoporosis was lower than the comparison group. The diagnosis of osteoporosis was obtained with the RAI-MDS 2.0 and the RAI-HC based on existing medical records or was self-reported, or reported by a family member, and that may underpin the underestimation of the prevalence of osteoporosis in the present study compared to other studies that diagnosed osteoporosis with bone densitometry techniques. Furthermore, osteoporosis is highly prevalent in the aging population and, considering that over 95% of the people in the comparison group were 45 years or older, that may explain the similar prevalence of osteoporosis between PwSCI and the comparison group across care settings. PwSCI reported more pathological fractures than the comparison group, in line with estimates from community-dwelling PwSCI compared to the general population [56,60]. However, while PwSCI have a risk of fracture risk 5- to 23-fold higher than age-matched persons without an SCI [60], the prevalence of fractures was lower in our population than in PwSCI living in the community. The RAI-MDS 2.0 collects data on pathological fractures that currently impact ADL, cognitive performance, mood and behaviour, medical treatment, nurse monitoring, or the risk of death. Therefore, all of the fractures that have healed, or that are no longer having an impact on such domains are not reported, and that explains the low prevalence of pathological fractures observed in PwSCI across the continuum of care.

The present study has several strengths. We performed a population-based, cross-sectional analysis including over 13,000 PwSCI, which represent over 10% of Canadian PwSCI, and that allows a broad generalization of our findings. interRAI systems provide population-level clinical data on PwSCI in four care settings in Ontario. However, it would be useful to consider the use of interRAI systems in sectors that do not currently use them in order to allow for comparability and interoperability of clinical data for PwSCI across their life course. Furthermore, while the incidence of SCI is higher in men, with a resulting underrepresentation of women in SCI research, almost 40% of our population was comprised of women, and the number of men and women in NHs and CCCs was very similar. The main clinical relevance of this study lies in the comprehensive and standardized nature of our assessments. In fact, interRAI assessment tools allowed us to perform a compelling evaluation of a large number of complications that may arise after a SCI, which informs clinical decisions and treatment plans. The present study highlights the complexity of SCI, thus prompting clinicians to go beyond the diagnosis of a SCI and take into account all of the consequences of SCI on physical and mental health when determining the priorities to address. Further, PwSCI are present in the different care settings, and this study demonstrated the variability of the characteristics of PwSCI across the continuum of care. interRAI has developed many assessment tools to conduct standardized assessments in the different care settings, and that allowed the comparison of the clinical characteristics of PwSCI across the continuum of care. Finally, one other strength of interRAI systems lies in the standardization of the assessments across several countries in the world. Indeed, the adoption of the interRAI assessment tools across countries allows population-level comparisons, and the opportunity to assess the effectiveness of different protocols and standards of care on several physical and psychosocial outcomes. This paper also presents a few limitations. First, the cross-sectional nature of this study does not allow to make inferences regarding the incidence of secondary complications and health conditions over time. Future studies should report on longitudinal data to explore whether the prevalence of secondary complications after an SCI changes over time. Second, data were collected in a subgroup of the SCI population across the continuum of care, and thus may not be representative of PwSCI without other chronic health conditions living in the community. Third, the interRAI instruments do not collect information on duration, level, and completeness of the SCI; therefore, we could not compare the prevalence of secondary complications across subgroups of people with SCI. Further, interRAI systems capture diagnoses regardless of whether they are reason for admission or not; therefore, it is not possible to determine if the health issues we detected were secondary complications of SCI or whether they were pre-existing or unrelated.

## 5. Conclusions

The clinical characteristics of PwSCI varied between care settings and PwSCI represent clinically distinct populations within each care setting. interRAI assessment tools allowed a comprehensive, multidimensional evaluation of the complexity of the health conditions following SCI, as well as a comparison of the clinical characteristics of PwSCI across the different care settings. A longitudinal analysis of the prevalence of heath conditions following SCI will provide further information with regard to the prevention and management of health conditions in PwSCI.

## Figures and Tables

**Table 1:** Sociodemographic characteristics for both PwSCI and the comparison group across the four care settings.

	Mental Health		Home Care		Nursing Home		Complex Continuing Care	
	Comparison Group	SCI	Comparison Group	SCI	Comparison Group	SCI	Comparison Group	SCI
**N**	312,401	32	966,381	312	724,256	5146	355,489	7646
**Female**	48.6%	34.4%	60.7%	32.1%	63.5%	47.1%	57.0%	45.3%
**Paraplegia** ^¶^	n/a	n/a	n/a	n/a	n/a	55.7%	n/a	50.7%
**Age Group**								
0–44	52.7%	40.6%	3.3%	22.1%	0.4%	7.4%	1.8%	13.2%
45–54	17.9%	25.0%	4.5%	12.8%	0.8%	9.0%	3.3%	13.5%
55–64	13.7%	21.9%	8.8%	23.7%	3.1%	16.1%	8.3%	18.4%
65–74	8.1%	12.5%	15.4%	18.0%	8.9%	21.2%	18.2%	22.9%
75–84	5.6%	0%	31.6%	16.4%	26.0%	24.6%	34.3%	21.0%
85+	2.1%	0%	36.5%	7.1%	60.9%	21.8%	34.1%	11.1%
**Married**								
Male	23.1%	28.6%	57.4%	42.9%	19.7%	15.9%	45.0%	30.2%
Female	27.6%	27.3%	28.4%	29.0%	7.2%	10.0%	23.9%	18.6%
Overall	25.3%	28.1%	39.8%	38.5%	11.8%	13.2%	33.0%	25.0%
Days of stay *								
0–30 days	99.8%	100.0%	32.2%	21.9%	20.0%	17.4%	79.5%	45.6%
31–90 days	0.1%	0.0%	11.2%	7.7%	4.1%	4.6%	1.8%	3.4%
90–365 days	0.0%	0.0%	19.2%	16.1%	22.0%	18.9%	13.7%	22.2%
366 and + days	0.1%	0.0%	37.4%	54.2%	53.9%	59.1%	5.0%	28.8%
**Emergency Department visits in the last 90 days ^¶^^¶^**								
None	n/a	n/a	76.5%	82.1%	85.0%	86.1%	56.7%	66.9%
1	n/a	n/a	16.7%	12.5%	12.7%	11.2%	34.8%	27.8%
2+	n/a	n/a	6.8%	5.5%	2.3%	2.7%	8.5%	5.3%
**Hospitalizations ^¶^** ^¶^								
None	n/a	n/a	64.8%	76.3%	86.9%	86.2%	35.5%	65.0%
1	n/a	n/a	29.0%	22.1%	11.7%	11.8%	46.2%	25.2%
2+	n/a	n/a	6.3%	1.6%	1.5%	2.0%	18.3%	9.9%
**Any Rehabilitation**								
Physical Therapy ^¶¶^	n/a	n/a	13.8%	13.8%	37.0%	45.1%	68.7%	55.6%
Occupational Therapy	22.6%	40.6%	16.3%	18.9%	5.3%	9.0%	56.8%	48.9%
Speech Language Pathology ^¶¶^	n/a	n/a	0.9%	1.0%	0.4%	0.9%	13.3%	12.4%
Social Work/Psychologist ^+^	60.1%	71.9%	1.8%	3.5%	0.8%	1.1%	13.5%	11.2%
Psychiatrist ^¶¶¶^	91.5%	93.8%	n/a	n/a	n/a	n/a	n/a	n/a
Other physician visits	59.3%	75.0%	33.8%	25.3%	69.2%	73.1%	94.9%	93.8%
Personal Support/Homemaking ^¶¶¶¶^	n/a	n/a	63.9%	69.6%	n/a	n/a	n/a	n/a
Recreation therapy ^¶¶¶¶¶^	33.7%	50.0%	n/a	n/a	13.8%	13.7%	32.0%	27.9%

* The within-setting comparison for MH was not statistically significant (*p* = 0.1556). ^+^ The within-setting comparison for MH was not statistically significant (*p* = 0.2821). ^¶^ Item available only in SCI group in NHs and CCCs. ^¶¶^ Item not available in MH. ^¶¶¶^ Item available only in MH. ^¶¶¶¶^ Item available only in HC. ^¶¶¶¶¶^ Item not available in HC.

**Table 2:** Comorbidities, medical interventions, and other clinical measures for both PwSCI and the comparison group across the four care settings.

	Mental Health		Home Care		Nursing Home		Complex Continuing Care	
	Comparison Group	SCI	Comparison Group	SCI	Comparison Group	SCI	Comparison Group	SCI
**N**	312,401	32	966,381	312	724,256	5146	355,489	7646
**CHESS Scale**								
0	67.3%	68.8%	20.8%	33.0%	29.1%	37.1%	17.2%	27.4%
1–2	29.5%	31.3%	56.0%	58.0%	48.8%	47.7%	44.3%	44.3%
3+	3.3%	0%	23.1%	9.0%	22.1%	15.2%	38.5%	28.3%
**Deafblind Severity Index** ^¶^								
0–1	n/a	n/a	79.1%	91.0%	65.9%	78.4%	80.6%	81.4%
2–4	n/a	n/a	20.6%	9.0%	33.2%	19.9%	18.9%	17.0%
5	n/a	n/a	0.3%	0%	0.9%	1.7%	0.5%	1.6%
**Diagnosis** ^¶^								
Heart Failure *	n/a	n/a	13.8%	5.1%	16.1%	8.0%	14.6%	6.7%
Emphysema/COPD	n/a	n/a	18.4%	10.9%	16.6%	11.7%	16.0%	9.0%
Diabetes ^†^	n/a	n/a	25.8%	18.6%	26.0%	26.8%	27.3%	24.4%
Cancer	n/a	n/a	19.3%	6.1%	11.4%	10.0%	28.7%	21.4%
Any psychiatric	n/a	n/a	34.3%	40.1%	54.1%	54.0%	40.1%	44.0%
Other CVD	n/a	n/a	67.5%	38.1%	65.6%	47.9%	60.5%	42.4%
**Health Issues**								
Unsteady gait ^¶^	n/a	n/a	67.9%	58.3%	27.4%	4.4%	46.8%	12.4%
Shortness of breath	3.1%	3.1%	31.2%	21.5%	13.1%	13.4%	26.5%	27.5%
Loss of appetite ^¶^	n/a	n/a	14.2%	8.3%	45.5%	35.9%	38.2%	26.7%
Pressure ulcers ^¶^	n/a	n/a	9.0%	26.9%	8.5%	14.2%	14.7%	21.7%
Trouble swallowing ^+^	2.5%	0%	11.2%	8.7%	29.7%	42.4%	26.0%	49.8%
Fair/Poor self-rated health	16.3%	33.3%	22.7%	18.9%	n/a	n/a	n/a	n/a
**Medical Interventions** ^¶^								
Chemotherapy ^§^	n/a	n/a	2.8%	0.3%	0.6%	0.9%	1.1%	1.6%
Dialysis ^‡^	n/a	n/a	1.6%	0.6%	0.9%	1.2%	2.1%	1.3%
Respirator/other device	n/a	n/a	0.7%	1.9%	0.2%	2.2%	0.5%	6.0%
Oxygen/Respiratory therapy	n/a	n/a	10.5%	9.0%	12.8%	14.1%	21.7%	31.4%
Intravenous	n/a	n/a	3.4%	1.0%	5.5%	8.2%	15.4%	19.5%
Nurse monitoring ^¶¶^	n/a	n/a	34.3%	43.0%	n/a	n/a	n/a	n/a
Wound care	n/a	n/a	14.6%	28.5%	27.0%	48.3%	42.0%	62.8%
**Occasional/worse Incontinence**								
Bladder	3.9%	18.8%	35.6%	26.0%	78.6%	63.4%	44.5%	45.1%
Bowel	2.7%	15.6%	15.1%	22.4%	59.2%	78.5%	37.8%	76.0%

* The differences in the prevalence of heart failure in PwSCI across settings was not statistically significant (*p* = 0.0067). ^†^ Prevalence of diabetes in PwSCI across sectors: *p* = 0.0003. ^+^ The within-sector difference for the MH cohort was not statistically significant (*p* = 0.6597). ^§^ Prevalence of PwSCI undergoing chemotherapy across sectors: *p* = 0.0004. ^‡^ No statistically significant differences in the prevalence of PwSCI needing dialysis were observed across care settings (*p* = 0.4345). ^¶^ Item not available in MHs. ^¶¶^ Item available only in HC.

**Table 3:** Pain, mobility, falls, fracture, ADL, and IADL for both PwSCI and the comparison group across the four care settings.

	Mental Health		Home Care		Nursing Home		Complex Continuing Care	
	Comparison Group	SCI	Comparison Group	SCI	Comparison Group	SCI	Comparison Group	SCI
**N**	312,401	32	966,381	312	724,256	5146	355,489	7646
**Pain Scale**								
0	77.8%	26.3%	33.4%	16.7%	58.3%	53.5%	27.5%	31.3%
1–2	20.2%	37.5%	51.8%	49.7%	38.6%	41.2%	64.2%	56.7%
3+	2.0%	6.3%	14.8%	33.7%	3.1%	5.3%	8.3%	12.1%
**Mobility**								
Uses wheelchair *	17.6%	31.3%	11.2%	60.6%	67.5%	86.0%	54.4%	70.0%
Walks independently	93.4%	75.0%	23.6%	9.3%	10.5%	0.8%	9.0%	1.1%
Bathes independently	n/a	n/a	20.1%	21.5%	0.6%	0.2%	3.2%	0.8%
**Falls and fractures**								
≥1 fall in last 90 days	4.9%	9.4%	38.2%	27.9%	19.1%	4.1%	28.0%	5.6%
Hip fracture ^¶+‡^	n/a	n/a	3.8%	1.3%	7.3%	3.2%	12.5%	3.1%
Pathological bone fracture ^¶¶^	n/a	n/a	n/a	n/a	1.3%	1.7%	2.6%	3.2%
Osteoporosis ^¶†^	n/a	n/a	21.0%	14.4%	24.3%	17.2%	14.6%	8.9%
**ADL Hierarchy Scale**								
0	86.4%	71.9%	49.4%	37.2%	2.5%	0.6%	4.7%	1.1%
1–2	7.4%	12.5%	29.5%	19.6%	10.3%	1.6%	21.1%	3.5%
3+	6.2%	15.6%	21.1%	43.3%	87.2%	97.8%	74.2%	95.4%
**IADL Items** ^¶¶¶^								
Any Impaired IADL	34.4%	34.4%	93.9%	94.0%	n/a	n/a	n/a	n/a
Meal Preparation	21.6%	31.3%	89.6%	90.1%	n/a	n/a	n/a	n/a
Managing Finances	18.9%	25.0%	72.5%	52.2%	n/a	n/a	n/a	n/a
Managing Medications	31.5%	31.3%	64.6%	46.2%	n/a	n/a	n/a	n/a
Transportation	17.8%	28.1%	80.4%	77.9%	n/a	n/a	n/a	n/a
**IADL Capacity Counts** ^¶¶¶^								
0	65.6%	65.6%	6.1%	6.4%	n/a	n/a	n/a	n/a
1	10.4%	3.1%	8.0%	12.2%	n/a	n/a	n/a	n/a
2	6.2%	3.1%	13.5%	24.4%	n/a	n/a	n/a	n/a
3	4.4%	6.3%	17.5%	2286%	n/a	n/a	n/a	n/a
4	13.4%	21.9%	54.9%	34.3%	n/a	n/a	n/a	n/a

* Between-groups difference in MH cohort: *p* = 0.0002. ^+^ The percentage of PwSCI who sustained a hip fracture was not statistically significant across sectors (*p* = 0.168). ^‡^ The difference in the prevalence of hip fractures between PwSCI and the comparison group in the HC cohorts was not statistically significant (*p* = 0.0194). ^†^ The difference in the prevalence of osteoporosis between PwSCI and the comparison group in the HC cohorts was not statistically significant (*p* = 0.0042). ^¶^ Item not available in MHs. ^¶¶^ Item not available in NHs and HC. ^¶¶¶^ Item not available in NHs and CCCs.

**Table 4:** Mental health and cognitive impairments, rehabilitation, and psychotropic medications.

	Mental Health		Home Care		Nursing Home		Complex Continuing Care	
	Comparison Group	SCI	Comparison Group	SCI	Comparison Group	SCI	Comparison Group	SCI
**N**	312,401	32	966,381	312	724,256	5146	355,489	7646
**Mental Health**								
Anxiety Symptoms *	56.7%	75.0%	20.5%	20.2%	30.6%	29.3%	25.2%	27.0%
Delirium	16.5%	18.8%	8.3%	2.6%	11.0%	8.3%	19.4%	15.6%
Any Aggressive Behaviour	21.1%	37.5%	11.6%	6.1%	42.7%	34.8%	26.1%	25.5%
Hallucinations/Delusions ^†^	29.0%	40.6%	5.5%	2.6%	9.8%	6.1%	7.6%	5.8%
**Depression Rating Scale ^§^**								
0	19.0%	13.0%	52.2%	50.0%	41.7%	44.4%	48.9%	45.4%
1–2	29.8%	31.3%	25.7%	25.0%	30.3%	30.2%	29.2%	29.3%
3+	51.3%	56.3%	22.1%	25.0%	28.0%	25.5%	21.9%	25.4%
**Communication Impairments**								
Expression	5.5%	0%	15.9%	3.5%	33.9%	33.3%	18.1%	34.4%
Comprehension ^¶^	n/a	n/a	17.6%	4.2%	35.5%	30.0%	20.5%	29.8%
**Cognitive Performance Scale**								
0	68.6%	62.5%	32.6%	56.1%	9.0%	26.1%	24.2%	27.8%
1–2	22.8%	28.1%	48.4%	40.7%	21.0%	24.1%	31.8%	22.2%
3–4	4.7%	6.3%	12.1%	2.2%	40.0%	21.1%	26.3%	16.3%
5–6	3.8%	3.1%	6.9%	1.0%	30.1%	28.8%	17.8%	33.8%
**Social and Economic Issues**								
Made economic trade-offs ^¶¶^	10.38%	12.50%	2.30%	6.41%	n/a	n/a	n/a	n/a
Conflict with others	16.3%	28.1%	14.1%	16.4%	9.5%	12.5%	11.3%	15.4%
Social Isolation	11.9%	3.1%	14.9%	18.3%	3.9%	5.3%	6.2%	7.2%
Caregivers distressed/overwhelmed ^¶¶^	37.0%	40.6%	30.7%	21.8%	n/a	n/a	n/a	n/a
**Physical Restraint Use**	5.7%	3.1%	0.8%	2.9%	10.0%	14.6%	11.7%	18.6%
**Psychotropic Drug Use** ^¶^								
Antipsychotics	n/a	n/a	10.9%	10.6%	28.0%	19.1%	21.1%	13.6%
Antidepressants	n/a	n/a	25.6%	38.8%	49.6%	48.8%	31.2%	32.9%
Anxiolytics	n/a	n/a	15.9%	20.8%	12.5%	17.8%	24.5%	26.0%
Sedatives	n/a	n/a	27.8%	34.9%	9.5%	12.8%	16.2%	14.5%

* Prevalence of anxiety symptoms in PwSCI across sectors: *p* = 0.0002. ^†^ The difference in hallucination/delusions were not statistically significant across sectors (*p* = 0.0351). ^§^ The differences in the Depression Rating Scale were not statistically significant across sectors (*p* = 0.2479). ^¶^ Item not available in MH. ^¶¶^ Item not available in NHs and CCCs.

## Data Availability

Data are available upon reasonable request.
